# Vaccination Hesitancy among Health-Care-Workers in Academic Hospitals Is Associated with a 12-Fold Increase in the Risk of COVID-19 Infection: A Nine-Month Greek Cohort Study

**DOI:** 10.3390/v14010026

**Published:** 2021-12-24

**Authors:** Fotinie Ntziora, Evangelia Georgia Kostaki, Ioannis Grigoropoulos, Amalia Karapanou, Ismini Kliani, Maria Mylona, Alexa Thomollari, Sotirios Tsiodras, Theoklis Zaoutis, Dimitrios Paraskevis, Nikolaos V. Sipsas, Anastasia Antoniadou, Petros P. Sfikakis

**Affiliations:** 1 1st Department of Propaedeutic Internal Medicine, University General Hospital Laiko, Medical School, National and Kapodistrian University of Athens, 11527 Athens, Greece; amkarapanou@gmail.com (A.K.); mamylona@gmail.com (M.M.); psfikakis@med.uoa.gr (P.P.S.); 2 Department of Hygiene, Epidemiology and Medical Statistics, Medical School, National and Kapodistrian University of Athens, 11527 Athens, Greece; ekostakh@med.uoa.gr (E.G.K.); dparask@med.uoa.gr (D.P.); 3 4th Department of Internal Medicine, University General Hospital Attikon, Medical School, National and Kapodistrian University of Athens, 12462 Athens, Greece; grigoropoulosioannis@gmail.com (I.G.); ismirnis@yahoo.gr (I.K.); athomollari@gmail.com (A.T.); tsiodras@med.uoa.gr (S.T.); ananto@med.uoa.gr (A.A.); 4 National Public Health Organization, 15123 Athens, Greece; t.zaoutis@eody.gov.gr; 5 Pathophysiology Department, University General Hospital Laiko, Medical School, National and Kapodistrian University of Athens, 11527 Athens, Greece; nsipsas@med.uoa.gr

**Keywords:** Coronavirus disease 2019 (COVID-19), vaccine, infection rate, hesitancy, Health-Care-Workers (HCWs)

## Abstract

Health-Care-Workers (HCWs) are considered at high risk for SARS-CoV-2 infection. We sought to compare rates and severity of Coronavirus disease 2019 (COVID-19) among vaccinated and unvaccinated HCWs conducting a retrospective cohort study in two tertiary Academic Hospitals, namely Laiko and Attikon, in Athens, Greece. Vaccinated by BNT162b2 Pfizer-BioNTech COVID-19 mRNA vaccine and unvaccinated HCWs were included and data were collected between 1 January 2021 and 15 September 2021. Overall, 2921 of 3219 HCWs without a history of Severe Acute Respiratory Syndrome Coronavirus-2 (SARS-CoV-2) infection were fully vaccinated during the study period (90.7% at each Hospital). Demographic characteristics were comparable between 102/2921 (3.5%) vaccinated and 88/298 (29.5%) unvaccinated HCWs with COVID-19, although age and occupation differed significantly. None were in need of hospital admission in the vaccinated Group, whereas in the unvaccinated Group 4/88 (4.5%) were hospitalized and one (1.1%) died. Multivariable logistic regression analysis revealed that lack of vaccination was an independent risk factor for COVID-19 with an odds ratio 11.54 (95% CI: 10.75–12.40). Vaccination hesitancy among HCWs resulted to highly increased COVID-19 rates; almost one in three unvaccinated HCWs was SARS-CoV-2 infected during the 9-month period. The absolute need of vaccination of HCWs, including boosting dose, is highlighted. Evidence should be used appropriately to overcome any hesitancy.

## 1. Introduction

Health-Care-Workers (HCWs) are at increased risk for Severe Acute Respiratory Syndrome Coronavirus-2 (SARS-CoV-2) infection worldwide through care and proximity to patients with Coronavirus disease 2019 (COVID-19) on top of the exposure risk through social interactions [1,2]. Additionally, HCWs have the potential to contribute to nosocomial transmission, although correct use of appropriate personal protective measures and consistency with good hand hygiene practices can minimize that risk. Vaccination of HCWs has been therefore prioritized as soon as the first COVID-19 vaccines have been made available. The mRNA vaccines are highly effective in fully vaccinated persons as demonstrated in several observational studies involving various populations, study designs and definitions [3]. Interim estimates of mRNA COVID-19 vaccine effectiveness in preventing SARS-CoV-2 infection among HCWs, first responders and other essential frontline workers were 90% for full immunization and 80% for partial immunization in a cohort study, thus confirming the findings of clinical trials among working-age adults in real-world conditions [4]. The mRNA COVID-19 vaccines have proven to be effective in preventing SARS-CoV-2 infection among populations at high risk, such as the HCWs, and a third dose of the vaccine seem to be boosting this effect [5,6,7].

Apart from the COVID-19 pandemic and despite the available scientific evidence, nowadays we must also face the so-called infodemic caused by misinformation and disinformation [8]. Vaccine hesitancy among HCWs has been recorded and common reasons for reporting intention to decline vaccination were inadequate information about the vaccines, followed by concerns about vaccine safety. Additionally, intention of HCWs not to get vaccinated was associated with not recommending COVID-19 vaccination to high-risk population [9]. Several approaches have been implemented so far in effort to overcome HCWs hesitancy, including interventions to raise awareness and knowledge. Recent evidence also suggests that the nocebo effect, referring to health changes attributed to negative expectations, may be involved in HCWs vaccine hesitancy, a challenge that needs to be addressed in a timely manner during the COVID-19 pandemic [10].

In Greece, as of December 2020 the mRNA COVID-19 vaccines have been available for vaccinating HCWs in priority, albeit on a voluntary basis. Since vaccine hesitancy is a well-established phenomenon, even among HCWs, as of 1 September 2021, vaccination has become mandatory by State law for all HCWs in our country. The aim of this study was to further investigate the impact of vaccine hesitancy in the rate and severity of COVID-19 in two Academic Hospitals in Athens, Greece.

## 2. Materials and Methods

### 2.1. Study Design

All HCWs with COVID-19 between 1 January 2021, and 15 September 2021, were prospectively recorded in an ongoing registry of two Academic Hospitals—namely Laiko and Attikon Hospitals—in Athens, Greece. This was a cohort study in which data from the two Hospital databases were analyzed retrospectively. During this period approximately up to 90 of 500 and up to 320 of 600 beds had been assigned to COVID-19 patients at Laiko and Attikon Hospitals, respectively. Data from 3219 HCWs in both hospitals were collected and analyzed to compare rates and severity of COVID-19 among vaccinated and unvaccinated individuals. Data with regards to distribution of patients among general (COVID-19, non-COVID-19) and intensive care (COVID-19, non-COVID-19) units during the study period can be found in the Supplementary Materials (Table S1).

### 2.2. Inclusion and Exclusion Criteria

All HCWs in the two Academic Hospitals were assessed for eligibility to be included in the study. Participants were excluded if they (i) had been vaccinated with a COVID-19 vaccine other than the BNT162b2 Pfizer-BioNTech COVID-19 mRNA vaccine, (ii) had received only one dose, (iii) were SARS-CoV-2 infected before the start of the study period (i.e., 1 January 2021) or the 1st vaccine dose, (iv) were temporarily transferred to other services/locations during the study period or had been on a long-term leave due to medical conditions, and (v) had been fully vaccinated (i.e., 7 days after they completed the two-dose vaccine series) after the end of the study period (i.e., 15 September 2021). A flow-chart of the steps followed for the participants’ selection applying the inclusion and exclusion criteria is provided in Table 1.

### 2.3. Case Definitions

As fully vaccinated were considered the HCWs who had received both doses of the BNT162b2 Pfizer-BioNTech COVID-19 mRNA vaccine at the time-point of 7 days after their second dose; HCWs who did not receive any COVID-19 vaccine dose during the study period were considered as unvaccinated. The date of infection was defined as the date at which the PCR test result became positive minus 5 days. SARS-CoV-2 infection was identified by a positive PCR test result during the study period. Fully vaccinated HCWs who had a positive PCR test result before their 2nd dose were considered as partially immunized were excluded and did not count as events (SARS-CoV-2 infected) in our analysis. A periodic pre-emptive infection control testing policy was applied by the Infection Control Committees of the two Academic Hospitals—COVID-19 PCR rhinopharyngeal test every 2 weeks at Laiko Hospital, self-reporting test weekly for vaccinated and PCR saliva test weekly for unvaccinated at Attikon Hospital. Both symptomatic and asymptomatic HCWs with a positive PCR test result were included in the study.

### 2.4. Data Collection

Data with regards to age, sex, occupation, comorbidities, immunosuppression, body mass index (BMI), exposure history, severity of symptoms if SARS-CoV-2 infected, hospital admission, and outcome were collected through personal interview of HCWs with COVID-19. Vaccination dates, time between 1st and 2nd vaccine dose, date of infection and time between immunization completion and infection were retrieved through the National COVID-19 Registry by authorized users.

### 2.5. Statistical Analysis

Demographic data were summarized using median and interquartile range (IQR) for the non-normally distributed continuous variables of our dataset, and absolute and relative frequencies for the categorical ones. Normality was examined by the Shapiro-Wilk test. Statistical analysis was carried out using the Mann–Whitney U test for the continuous non-normally distributed variables and the Pearson’s chi-squared test as well as Fisher’s exact test for the categorical variables. In addition, a multivariable logistic regression model was fit to a subset of the original data (*n* =3219), consisting of 3216 complete observations. In this model we used the SARS-CoV-2 infection in HCWs participated in our study as the binary dependent variable (outcomes: infected/non-infected) and age, gender, occupation, vaccination and Academic Hospital as possible explanatory (independent) variables”. Clustered standard errors at the hospital level were used in the model to take into account and adjust for potential within-cluster correlation. The level of significance was set at 0.05. Analysis was performed on STATA 16.0 (StataCorp. 2019. Stata Statistical Software: Release 16. College Station, TX: StataCorp LLC).

## 3. Results

### 3.1. Study Population

During the study period (1 January 2021–15 September 2021), a total of 3806 HCWs from two Academic Hospitals (1661 and 2145 from Laiko and Attikon Hospitals, respectively) were evaluated for eligibility to be included in the study. Following the application of exclusion criteria, a total of 3219 HCWs (1436 and 1783 from Laiko and Attikon Hospitals, respectively) were eligible and included in the analysis (Table 1). Among participants, physicians were more prone to be vaccinated (1058/1086; 97.4%) [461/471 (97.9%) and 597/615 (97.1%) at Laiko and Attikon Hospitals, respectively], followed by nurses (1031/1153; 89.4%) [468/524 (89.3%) and 563/629 (89.5%) at Laiko and Attikon Hospitals, respectively], and all the other HCWs (832/980; 84.9%) [374/441 (84.8%) and 458/539 (85%) at Laiko and Attikon Hospitals, respectively], who displayed the higher rates of hesitancy (*p*-value < 0.001).

### 3.2. Population Characteristics

The demographic and clinical characteristics of the 3219 enrolled participants are summarized in Table 2. Overall, 2921 of 3219 (90.7%) HCWs were COVID-19 vaccinated during the study period [1303/1436 (90.7%) at Laiko and 1618/1783 (90.7%) at Attikon Hospital]. Among them, 102 (3.5%) were SARS-CoV-2 infected. The proportion of SARS-CoV-2 infected among the unvaccinated HCWs was 29.5% (88/298). Demographic characteristics of SARS-CoV-2 infected HCWs were similar between the vaccinated and unvaccinated Group except from age, which was lower in the vaccinated Group (39 vs. 45 years) (*p*-value = 0.0012), and occupation. Even among SARS-CoV-2 infected participants, physicians had the highest vaccination rate 33/35 (94.3%), followed by nurses 45/89 (50.6%), and other HCWs 24/66 (36.4%) (*p*-value < 0.001). Underlying medical conditions were present in 23/102 (22.5%) and 22/88 (25%) of the vaccinated and unvaccinated HCWs, respectively. Immunosuppression was present in 4/102 (3.9%) of the vaccinated and 1/88 (1.1%) of the unvaccinated HCWs. The median time in days between the two COVID-19 vaccine doses was similar for the two Academic Hospitals and in accordance with the national guidelines on vaccination (i.e.,an interval of 21 days between the two vaccine doses). The median time between immunization and SARS-CoV-2 infection was 91 days, ranging from 55 to 169 days.

### 3.3. Outcomes

**Figure 1:**
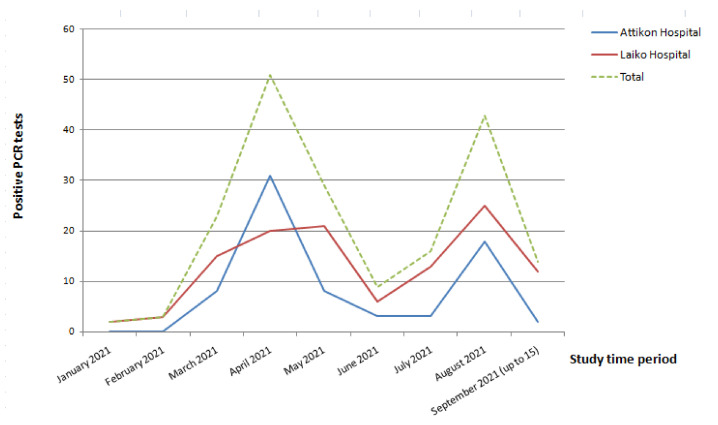
Severe Acute Respiratory Syndrome Coronavirus-2 (SARS-CoV-2) infection rate per month among vaccinated and unvaccinated Health-Care-Workers (HCWs) in two Academic Hospitals in Athens, Greece, during the study period.

The main outcomes of our study are summarized in Table 3 and Table 4. Among vaccinated HCWs, symptomatic COVID-19 was noted in 78 of 102 (76.5%) compared to 62 of 88 (70.5%) in the unvaccinated Group. None was admitted to the hospital due to COVID-19 severity and none died in the vaccinated Group. In contrast, in the unvaccinated Group 4 of 88 (4.5%) required hospitalization and 1 (1.1%) died. Multivariable logistic regression analysis revealed that vaccination hesitancy was independently associated with SARS-CoV-2 infection (odds ratio 11.54, 95% CI: 10.75–12.40; *p*-value < 0.001) (Table 4). Different working environment was also found to be associated with increased risk of SARS-CoV-2 infection, as HCWs at Laiko Hospital had a two-fold increase in the risk of infection compared to those working at Attikon Hospital. SARS-CoV-2 infection rate among vaccinated and unvaccinated HCWs in the two Academic Hospitals during the study period are shown in Figure 1. The two waves seen on the graph are in consistency with the incidence of COVID-19 in the general population and can be attributed to the B.1.17 “alpha” variant that in February 2021 became dominant in Greece and the B.1.617.2 “delta” variant that was accumulated in July 2021 according to the National Surveillance System data [11].

## 4. Discussion

Our study supports the protective effect of vaccination in the overall risk of COVID-19 among HCWs. In the context of our study, the odds ratio approximates to the relative risk. Therefore, the risk of being SARS-CoV-2 infected was almost 12 times higher among unvaccinated than vaccinated HCWs. Our study is not without limitations. Data from HCWs that were fully vaccinated with two doses of the BNT162b2 Pfizer-BioNTech COVID-19 mRNA vaccine were analyzed and therefore our conclusions apply to this particular type of vaccine. The difference in the sample size between the vaccinated and unvaccinated HCWs included in our study from the two Academic Hospitals was nearly tenfold, which may have an impact on the results balance. However, since the total number of HCWs per Hospital was included and as of 1 September 2021, vaccination has become mandatory by State law for all HCWs in our country, there is no way to increase the sample size of the unvaccinated Group. Strengths of the study include a long follow-up period in a health care setting with participants at high risk of infection through working environment on top of any social accountings and interactions. Moreover, the two studied Groups of vaccinated and unvaccinated HCWs with COVID-19 showed no major differences in terms of demographics and clinical characteristics.

Recent studies from other countries are in accordance with our findings. The SIREN study, a prospective multicentre cohort study that was held in UK among 23,324 HCWs, showed that during a 2-month period following completion of the vaccination program with BNT162b2 Pfizer-BioNTech COVID-19 mRNA vaccine, symptomatic and asymptomatic infections occurred in 80 (3.8%) among vaccinated and 977 (38%) among unvaccinated HCWs [1]. On the other hand, Victor et al. showed that among 10,600 HCWs in a 2600-bed tertiary care hospital in India, 7080 (66.8%) of whom fully vaccinated against COVID-19, the risk of infection was lower when compared with unvaccinated HCWs, albeit with a relatively small risk ratio of 0.35 (95% CI: 0.32–0.39). The protective effect of this vaccine type in preventing infection, hospitalization, need for high-flow oxygen, and intensive care unit admission was 65%, 77%, 92%, and 96%, respectively [12]. COVID-19 vaccination not only reduced the hospital admission rate and mortality, but also prevented seven out of ten episodes of absenteeism—i.e., absence of a HCWs from work duties due to onset of symptoms for isolation purposes following exposure to SARS-CoV-2, quarantine—among HCWs, as well as significantly reduced the length of absence from work and therefore the need for increased workload due to emergency healthcare demands for the rest of the staff [13]. Experience through studies coming from Israel, the first country to have completed vaccination for a considerable percentage of all individuals, including HCWs, early on inthe pandemic has an added value showing the protective effect of the COVID-19 vaccine as well as that of subsequent booster doses [14,15,16].

The existing COVID-19 vaccines have shown efficacy in the real-world setting even in populations with underlying medical conditions, such as diabetes mellitus, chronic lung disease, chronic cardiovascular diseases, and increased BMI [17]. On the other hand, it is well described that COVID-19 vaccine effectiveness is significantly reduced for patients with immunocompromising conditions, especially patients with active solid organ or hematologic malignancy or solid organ transplantation [18]. Published data support that COVID-19 vaccine’ effectiveness remains at a protective level even when novel COVID-19 variants of interest are isolated. In a prospective cohort study among SARS-CoV-2 infected vaccinated HCWs, sequencing of 50 vaccine breakthrough infections showed an overall genome divergence compared to the original Wuhan-Hu-1 sequence, as expected, however no clear sign of increased or greater divergence in the spike protein, compared to time-matched, unvaccinated control sequences was noted [19]. Had patients at increased risk for severe COVID-19 been vaccinated, many of the COVID-19 related hospital admissions and deaths might have been avoided [20].

Although several studies support the effectiveness of the COVID-19 vaccines for the prevention of SARS-CoV-2 infection and several reports have analyzed in depth the link between COVID-19 vaccination and potential occurrence of side effects [5,6,7], COVID-19 vaccine hesitancy among HCWs seems to be linked to a higher susceptibility to nocebo effects. Factors known to influence nocebo effects include personality characteristics, previous positive or negative medical experiences, social observational learning, negative conditioning, excessive anxiety and other mood disorders, and the physician’s communication skills. Media or internet information is also essential in modifying placebo or nocebo effects and therefore a campaign to address nocebo-prone people might be of benefit to overcome COVID-19 vaccine hesitancy [10]. A recent study investigating rates of intention to get vaccinated against COVID-19 among HCWs in Greece revealed that the probability of intending to get vaccinated increased with male gender, being a physician, history of complete vaccination against hepatitis B, history of vaccination against pandemic A (H1N1) in 2009–2010, belief that COVID-19 vaccination should be mandatory for HCWs, and increased confidence in vaccines in general during the COVID-19 pandemic. On the other hand, factors associated with vaccine hesitancy included no vaccination against influenza the past season, no intention to get vaccinated against influenza in 2020–2021, and no intention to recommend COVID-19 vaccination to high-risk patients [9]. Our study showed that apart from COVID-19 vaccination, other factors such as age, occupation and work location had an additional impact on risk of SARS-CoV-2 infection.

Using case-based surveillance data reported to the European Surveillance System (TESS) by nine European countries, a study by Ferland et al. showed that the adjusted risk of COVID-19 requiring hospitalization or admission to ICU was respectively 1.8 and 1.9 times higher in HCWs than in non-HCWs (95% CIs 1.2–2.7 and 1.1–3.2, respectively), although the adjusted risk of death did not differ significantly [2]. A recent upturn of SARS-CoV-2 infection rate mainly attributed to the “delta” variant and more recently to the “omicron” variant, has led some countries’ governments to mandate employees at high risk of COVID-19 to be vaccinated. In countries such as Australia, Britain, Canada, Costa Rica, Fiji, France, Greece, Hungary, Indonesia, Italy, Kazakhstan, Micronesia, Russia, Saudi Arabia, Tasmania, and Turkmenistan COVID-19 vaccination is mandatory for those at high risk, including the HCWs. In contrast, many countries in Europe do not have the COVID-19 vaccine mandatory for HCWs, as ethical issues rise and discussions on human rights are ongoing [21]. A strong anti-vaccine wave has emerged, and multi-sectorial approach might be necessary to address this challenge. Media or internet information is also essential in behavior modification and might be of benefit to overcome COVID-19 vaccine hesitancy [22,23].

Especially among HCWs, apart from the individual benefit of preventing severe COVID-19 and death, reducing the risk of SARS-CoV-2 infection can further prevent secondary transmission especially from asymptomatic stage in the Hospital setting [4,24]. Moreover, vaccinated people with breakthrough infections, including the “delta” variant, are less likely to develop symptoms in particular severe symptoms, are more likely to recover from their illness quickly and much less likely to require hospitalization or to transmit the infection to others [25,26,27]. The implication of additional preventive measures, such as COVID-19 vaccine booster doses and continued masking in particular for individuals at highest risk for breakthrough infections, may facilitate control of COVID-19 [18]. We should use what we—the health care providers—know best, i.e., evidence, science, and care for the others, to overcome any hesitancy that may rise.

## 5. Conclusions

The BNT162b2 Pfizer-BioNTech COVID-19 mRNA vaccine was highly effective under real-world conditions in preventing SARS-CoV-2 infection among people at increased risk of exposure as HCWs, even when additional risk factors were present, such as immunosuppression. Vaccination hesitancy among HCWs was associated with an almost 12-fold increase in the risk of SARS-CoV-2 infection, whereas almost 1 in 3 unvaccinated HCWs in Academic Hospitals were SARS-CoV-2 infected during a 9-month period. The long-term protective effect of the COVID-19 vaccines is not yet well known, however, according to our findings the protective effect of the COVID-19 vaccine lasted for at least 9 months post immunization. These results highlight the necessity for HCWs vaccination, including any additional boosting dose. Appropriate evidence-based approaches should help to overcome any hesitancy.

## Figures and Tables

**Table 1:** Participants’ selection among Health-Care-Workers (HCWs) in two Academic Hospitals in Athens, Greece.

Participants’ Selection According to Exclusion Criteria	Number of Evaluated Participants (*n*)	
Total HCWs evaluated	3806	
Total HCWs evaluated per hospital	Laiko Hospital 1661	Attikon Hospital 2145
Vaccinated with other than BNT162b2 Pfizer- BioNTech COVID-19 mRNA vaccine	63	110
Vaccinated only with 1 dose	36	94
Fully vaccinated after the end of the study period	56	67
SARS-CoV-2 infected before the start of the study period	59	88
SARS-CoV-2 infected before the 1st dose	11	3
Total HCWs included at the analysis per hospital	1436	1783
Total HCWs included in the analysis (study population)	3219	

COVID-19: Coronavirus disease 2019; HCWs: Health-Care-Workers; SARS-CoV-2: Severe Acute Respiratory Syndrome Coronavirus-2.

**Table 2:** Demographic and clinical characteristics of vaccinated and unvaccinated Health-Care-Workers (HCWs) with COVID-19.

Variables	Laiko Hospital *n* = 1436		Attikon Hospital *n* = 1783		Total *n* = 3219		*p*-Value
Vaccination	Vaccinated 1303 (90.7%)	Unvaccinated 133 (9.3%)	Vaccinated 1618 (90.7%)	Unvaccinated 165 (9.3%)	Vaccinated 2921 (90.7%)	Unvaccinated 298 (9.3%)	
SARS-CoV-2 infected							
Vaccination	Vaccinated 66 (5.1%)	Unvaccinated 51 (38.4%)	Vaccinated 36 (2.2%)	Unvaccinated 37 (22.4%)	Vaccinated 102 (3.5%)	Unvaccinated 88 (29.5%)	
Demographics of SARS-CoV-2 infected							
Vaccination	Vaccinated	Unvaccinated	Vaccinated	Unvaccinated	Vaccinated	Unvaccinated	
Sex	F: 48 (72.7%) M: 18 (27.3%)	F: 34 (66.7%) M: 17 (33.3%)	F: 26 (72.2%) M: 10 (27.8%)	F: 34 (91.9%) M: 3 (8.1%)	F: 74 (72.6%) M: 28 (27.4%)	F: 68 (77.3%) M: 20 (22.7%)	0.455 *
Median age (in years; IQR)	39 (31, 50)	48 (38, 56)	41 (32, 49)	44 (39, 50)	39 (31, 50)	45 (38, 53)	0.0012 ***
BMI	Underweight: 3 (4.5%) Normal: 44 (66.7%) Overweight: 14 (21.2%) Obese: 4 (6.1%) Extremely obese: 1 (1.5%)	Underweight: 4 (7.8%) Normal: 34 (66.7%) Overweight: 9 (17.7%) Obese: 4 (7.8%) Extremely obese: 0 (0%)	Underweight: 0 (0%) Normal: 28 (77.8%) Overweight: 5 (13.9%) Obese: 0 (0%) Extremely obese: 0 (0%) Unknown: 3 (8.3%)	Underweight: 0 (0%) Normal: 28 (75.7%) Overweight: 7 (18.9%) Obese: 0 (0%) Extremely obese: 0 (0%) Unknown: 2 (5.4%)	Underweight: 3 (2.9%) Normal: 72 (70.6%) Overweight: 19 (18.7%) Obese: 4 (3.9%) Extremely obese: 1 (1.0%) Unknown: 3 (2.9%)	Underweight: 4 (4.5%) Normal: 62 (70.5%) Overweight: 16 (18.2%) Obese: 4 (4.5%) Extremely obese: 0 (0%) Unknown: 2 (2.3%)	0.990 **
Occupation	Doctor: 23 (34.9%) Nurse: 28 (42.4%) Other: 15 (22.7%)	Doctor: 1 (2.0%) Nurse: 23 (45.1%) Other: 27 (52.9%)	Doctor: 10 (27.8%) Nurse: 17 (47.2%) Other: 9 (25.0%)	Doctor: 1 (2.7%) Nurse: 19 (51.3%) Other: 17 (46.0%)	Doctor: 33 (32.4%) Nurse: 45 (44.1%) Other: 24 (23.5%)	Doctor: 2 (2.3%) Nurse: 44 (50%) Other: 42 (47.7%)	<0.001*
Comorbidities							
Present Yes	14 (21.2%)	12 (23.5%)	9 (25.0%)	10 (27.0%)	23 (22.5%)	22 (25.0%)	0.692 *
No	52 (78.8%)	39 (76.5%)	27 (75.0%)	27 (73.0%)	79 (77.5%)	66 (75.0%)	
Immunosuppression Yes	3 (4.6%)	1 (2.0%)	1 (2.8%)	0 (0%)	4 (3.9%)	1 (1.1%)	0.375 **
No	63 (95.4%)	50 (98.0%)	35 (97.2%)	37 (100%)	98 (96.1%)	87 (98.9%)	
Exposure HxYes	35 (53.0%)	19 (37.3%)	2 (5.6%)	4 (10.8%)	37 (36.3%)	23 (26.1%)	0.134 *
No	31 (47.0%)	32 (62.7%)	34 (94.4%)	33 (89.2%)	65 (63.7%)	65 (73.9%)	
Time							
Median time between the 2 doses (in days; IQR)	21 (21, 22)		21.5 (21, 23)		21 (21, 22)		-
Median time between immunity achieved and SARS-CoV-2 infection (in days; IQR)	136 (55, 177)		74 (53.5, 138.5)		91 (55, 169)		-

* Pearson’s chi-squared test; ** Fisher’s exact test; *** Mann–Whitney U test. BMI: Body Mass Index; Hx: History; IQR: Interquartile range; SARS-CoV-2: Severe Acute Respiratory Syndrome Coronavirus-2.

**Table 3:** Main outcomes among SARS-CoV-2 infected vaccinated and unvaccinated Health-Care-Workers (HCWs) in two Academic Hospitals in Athens, Greece, in terms of infection, hospital admission and death.

Variables	Laiko Hospital		Attikon Hospital		Total		*p*-Value
SARS-CoV-2 Infected							
Vaccination	**Vaccinated** **66 (5.1%)**	**Unvaccinated** **51 (38.4%)**	**Vaccinated** **36 (2.2%)**	**Unvaccinated** **37 (22.4%)**	**Vaccinated** **102 (3.5%)**	**Unvaccinated** **88 (29.5%)**	
Symptomatic Yes	48 (72.7%)	29 (56.9%)	30 (83.3%)	33 (89.2%)	78 (76.5%)	62 (70.5%)	0.348 *
No	18 (27.3%)	22 (43.1%)	6 (16.7%)	4 (10.8%)	24 (23.5%)	26 (29.5%)	
Hospital admission Yes	0 (0%)	3 (5.9%)	0 (0%)	1 (2.7%)	0 (0%)	4 (4.5%)	0.044 **
No	66 (100%)	48 (94.1%)	36 (100%)	36 (97.3%)	102 (100%)	84 (95.5%)	
OutcomeDeath	0 (0%)	1 (2.0%)	0 (0%)	0 (0%)	0 (0%)	1 (1.1%)	0.463 **
Cure	66 (100%)	50 (98.0%)	36 (100%)	37 (100%)	102 (100%)	87 (98.9%)	

* Pearson’s chi-squared test; ** Fisher’s exact test. SARS-CoV-2: Severe Acute Respiratory Syndrome Coronavirus-2.

**Table 4:** Multivariable logistic regression estimates using the SARS-CoV-2 infection as the binary outcome variable (*n* = 3216).

Explanatory Variable	Odds Ratio	95% Confidence Interval (CI)	*p*-Value
Age (in years)	0.975	0.973–0.976	<0.001
Gender			
Male	reference category		
Female	1.11	0.93–1.33	0.255
Occupation			
Doctor	reference category		
Nurse	1.89	1.33–2.67	<0.001
Other	1.44	1.27–1.63	<0.001
Vaccination			
Vaccinated	reference category		
Unvaccinated	11.54	10.75–12.40	<0.001
Hospital			
Attikon	reference category		
Laiko	2.30	2.25–2.35	<0.001

## Data Availability

The data presented in this study are available on request from the corresponding author. The data are not publicly available in accordance to General Data Protection Regulation (GDPR).

## Supplementary Materials

The following are available online at https://www.mdpi.com/article/10.3390/v14010026/s1, Table S1: Data with regards to distribution of patients among General (COVID-19, non-COVID-19) and Intensive Care (COVID-19, non-COVID-19) Units during the study period in two Academic Hospitals, Athens Greece, expressed as numbers of patients (patient-days)
