# Supplementary material for: Vaccination Hesitancy among Health-Care-Workers in Academic Hospitals Is Associated with a 12-Fold Increase in the Risk of COVID-19 Infection: A Nine-Month Greek Cohort Study
Source: Viruses. 2021 Dec 24;14(1):26. doi: 10.3390/v14010026 (PMC8779273; doi:10.3390/v14010026)
Supplement: Supplementary file 1 [file viruses-14-00026-s001.zip › viruses-1501965-supplementary.pdf]

viruses

MDPI

Supplementary Materials

Vaccination Hesitancy among Health-Care-Workers in Academic Hospitals Is Associated With a 12-Fold Increase in the Risk of COVID-19-19 Infection: A Nine-Month Greek Cohort Study

Table S1. Data with regards to distribution of patients among General (COVID-19, non-COVID-19) and Intensive Care (COVID-19, non-COVID-19) Units during the study period in two Academic Hospitals, Athens Greece, expressed as numbers of patients (patient-days).

Hospital Admissions/Month	Laiko Hospital *n*= 41,721 (124,025)		Attikon Hospital *n*= 21,675 (127,573)		Total *n*= 63,396 (251,598)	
January 2021	non-COVID-19 *n*= 4084 (12,812) ICU *n*= 63 (511)	COVID-19 *n*= 57 (312) ICU *n*= 0 (0)	non-COVID-19 *n*= 2173 (13,679) ICU *n*= 47 (476)	COVID-19 *n*= 114 (823) ICU *n*= 17 (235)	non-COVID-19 *n*= 6257 (26,491) ICU *n*= 110 (987)	COVID-19 *n*= 171 (1135) ICU *n*= 17 (235)
February 2021	non-COVID-19 *n*= 4187 (11,521) ICU *n*= 84 (552)	COVID-19 *n*= 301 (1158) ICU *n*= 0 (0)	non-COVID-19 *n*= 2165 (12,280) ICU *n*= 54 (367)	COVID-19 *n*= 284 (1770) ICU *n*= 49 (416)	non-COVID-19 *n*= 6352 (23,801) ICU *n*= 138 (919)	COVID-19 *n*= 585 (2928) ICU *n*= 49 (416)
March 2021	non-COVID-19 *n*= 4416 (11,153) ICU *n*= 91 (637)	COVID-19 *n*= 538 (2362) ICU *n*= 0 (0)	non-COVID-19 *n*= 1851 (9089) ICU *n*= 17 (125)	COVID-19 *n*= 689 (4756) ICU *n*= 97 (1150)	non-COVID-19 *n*= 6267 (20,242) ICU *n*= 108 (762)	COVID-19 *n*= 1227 (7118) ICU *n*= 97 (1150)
April 2021	non-COVID-19 *n*= 4137 (9986) ICU *n*= 83 (602)	COVID-19 *n*= 539 (2876) ICU *n*= 10 (71)	non-COVID-19 *n*= 1734 (8188) ICU *n*= 19 (142)	COVID-19 *n*= 724 (5320) ICU *n*= 99 (1146)	non-COVID-19 *n*= 5871 (18,174) ICU *n*= 102 (744)	COVID-19 *n*= 1263 (8196) ICU *n*= 102 (744)
May 2021	non-COVID-19 *n*= 4216 (11,261) ICU *n*= 77 (619)	COVID-19 *n*= 265 (1507) ICU *n*= 18 (114)	non-COVID-19 *n*= 1927 (10,611) ICU *n*= 39 (276)	COVID-19 *n*= 474 (3761) ICU *n*= 69 (931)	non-COVID-19 *n*= 6143 (21,872) ICU *n*= 116 (895)	COVID-19 *n*= 739 (5268) ICU *n*= 87 (1045)
June 2021	non-COVID-19 *n*= 4360 (12,595) ICU *n*= 70 (563)	COVID-19 *n*= 123 (687) ICU *n*= 0 (0)	non-COVID-19 *n*= 2346 (13,475) ICU *n*= 53 (368)	COVID-19 *n*= 170 (856) ICU *n*= 36 (504)	non-COVID-19 *n*= 6706 (26,070) ICU *n*= 123 (931)	COVID-19 *n*= 293 (1543) ICU *n*= 36 (504)
July 2021	non-COVID-19 *n*= 4820 (13,828) ICU *n*= 68 (515)	COVID-19 *n*= 138 (612) ICU *n*= 0 (0)	non-COVID-19 *n*= 2382 (13,731) ICU *n*= 52 (326)	COVID-19 *n*= 139 (725) ICU *n*= 20 (295)	non-COVID-19 *n*= 7202 (27,559) ICU *n*= 120 (841)	COVID-19 *n*= 277 (1337) ICU *n*= 20 (295)
August 2021	non-COVID-19 *n*= 3851 (11,509) ICU *n*= 66 (544)	COVID-19 *n*= 145 (1087) ICU *n*= 0 (0)	non-COVID-19 *n*= 2003 (11,773) ICU *n*= 32 (326)	COVID-19 *n*= 263 (1734) ICU *n*= 32 (364)	non-COVID-19 *n*= 5854 (23,282) ICU *n*= 98 (870)	COVID-19 *n*= 408 (2821) ICU *n*= 32 (364)
September 2021	non-COVID-19 *n*= 4691 (12,803) ICU *n*= 76 (477)	COVID-19 *n*= 139 (715) ICU *n*= 8 (36)	non-COVID-19 *n*= 1323 (6263) ICU *n*= 27 (205)	COVID-19 *n*= 131 (830) ICU *n*= 24 (257)	non-COVID-19 *n*= 6014 (19,066) ICU *n*= 103 (682)	COVID-19 *n*= 270 (1545) ICU *n*= 32 (293)

COVID-19: Coronavirus disease 2019; ICU: Intensive Care Unit.
